# A Novel Chronotype-Based Mediterranean Diet Pyramid: An Updated Representation by the Italian Society of Endocrinology (SIE) and the Italian Society of Dietetics and Clinical Nutrition (ADI)

**DOI:** 10.1007/s13668-026-00731-x

**Published:** 2026-02-13

**Authors:** Luigi Barrea, Ludovica Verde, Elisabetta Camajani, Giuseppe Annunziata, Maria Pina Mollica, Marianna Minnetti, Giovanna Trinchese, Pietro Losignore, Annamaria Colao, Alberto Ferlin, Carmela Bagnato, Diego Ferone, Barbara Paolini, Massimiliano Caprio, Giovanna Muscogiuri

**Affiliations:** 1 Dipartimento Di Psicologia E Scienze Della Salute, Centro Direzionale, Università Telematica Pegaso, Via Porzio, Isola F2, Naples, 80143 Italy; 2 Department of Medicine, Division of Endocrinology, University of Arizona, Tucson, AZ 85724 USA; 3 Dipartimento di Medicina Clinica e Chirurgia, Centro Italiano per la cura e il Benessere del Paziente con Obesità (C.I.B.O), Università degli Studi di Napoli Federico II, Via Sergio Pansini 5, Naples, 80131 Italy; 4 Laboratory of Cardiovascular Endocrinology, IRCCS San Raffaele, Rome, Italy; 5 Department for the Promotion of Human Sciences and Quality of Life, San Raffaele Roma Open University, Rome, Via di Val Cannuta 247, 00166 Italy; 6 Department of Biology, University of Naples Federico II, via Cintia 21, 80126 Naples, Italy; 7 Department of Experimental Medicine, Sapienza University, Rome, Italy; 8 UNESCO Chair on Urban Health Education and Research for Improved Health and Wellbeing in the Cities, Sapienza University, Rome, Italy; 9 UOSD Clinical Nutrition and Dietetic, Hospital Matera, Matera, 75100 Italy; 10 Dipartimento Di Medicina Clinica E Chirurgia, Unità di Endocrinologia, Diabetologia ed Andrologia, Università Degli Studi Di Napoli Federico II, Naples, Italy; 11 Cattedra Unesco Educazione Alla Salute E Allo Sviluppo Sostenibile, University Federico II, Naples, Italy; 12 Department of Systems Medicine, Unit of Andrology and Reproductive Medicine, University Hospital of Padova, Padova, 35122 Italy; 13 Department of Internal Medicine and Medical Specialties, School of Medical and Pharmaceutical Sciences, Endocrinology Unit, University of Genova, Genoa, 16132 Italy; 14 UOSA of Dietetics and Clinical Nutrition, Azienda Ospedaliera Universitaria Senese, Policlinico Santa Maria Alle Scotte, Siena, Italy; 15 Department of Medicine, University of Padova, 35122 Padova, Italy

**Keywords:** Mediterranean diet pyramid, Chronotype, Chrononutrition, Human health, Obesity

## Abstract

**Purpose of Review:**

This review explores the integration of chronobiology and chronotype-specific factors into the Mediterranean Diet (MD) framework. While the MD is widely recognized for its cardiometabolic and neuroprotective effects, current guidelines do not address the timing of food intake or the influence of individual chronotype. Given the growing evidence linking circadian alignment, meal timing, and metabolic health, we propose a revised Mediterranean Diet pyramid tailored to chronotype and lifestyle determinants, herein referred to as the chronotype-based Mediterranean Diet Pyramid of the Italian Society of Endocrinology (SIE) and the Italian Society of Dietetics and Clinical Nutrition (ADI).

**Recent Findings:**

Chrononutrition emphasizes the timing, frequency, and regularity of meals as key elements for metabolic regulation. Early-day energy intake, time-restricted eating, and alignment with circadian rhythms improve glycemic control, weight management, and cardiovascular outcomes. Morning chronotypes tend to show higher adherence to the MD, whereas evening chronotypes more frequently delay meals, skip breakfast, and consume energy-dense foods at night, contributing to poorer metabolic profiles. Sleep duration and quality further modulate appetite regulation and dietary adherence. Building on these insights, the proposed pyramid integrates circadian cues, represented by sun and moon symbols, to guide nutrient timing. Foods rich in carbohydrates and fiber (whole grains, legumes, fruits) are emphasized earlier in the day, while protein- and vegetable-based meals are prioritized in the evening, alongside sleep-promoting foods such as dairy, nuts, and seeds. Lifestyle pillars, including physical activity, adequate sleep, and the alignment of eating schedules with chronotype, form the foundation of the pyramid.

**Summary:**

The integration of chronobiological principles into the MD offers a novel paradigm that couples dietary quality with circadian alignment. A chronotype-oriented MD pyramid may enhance adherence, optimize metabolic flexibility, and reinforce the MD as a holistic model encompassing nutrition, lifestyle, and sustainability.

## Introduction

Circadian rhythms, governed by central and peripheral clocks, regulate behavioral and metabolic functions across the 24-hour cycle [1–3]. Feeding acts as a major zeitgeber, and nutrient timing can either synchronize or desynchronize peripheral oscillators with the central pacemaker [4, 5], thereby influencing hormone secretion and metabolic homeostasis [6–8]. Early-day food intake appears metabolically advantageous, while late or irregular meals are associated with higher inflammatory and metabolic biomarkers and poorer weight-loss outcomes [8–10]. The individual expression of circadian rhythmicity is expressed by chronotype, which shapes variability in sleep–wake cycles and eating behaviors [11, 12]. Morning chronotypes show phase-advanced rhythms, whereas evening chronotypes display delayed patterns, greater social jet lag, and more irregular eating habits [13–17]. An evening preference correlates with higher consumption of energy-dense foods, caffeinated and alcoholic beverages, and lower fruit and vegetable intake, contributing to an increased risk of obesity, metabolic dysfunction, and psychiatric symptoms [18–23]. In this context, chrononutrition integrates circadian biology into dietary strategies [24], focusing on core dimensions such as timing, frequency, and regularity of eating [25, 26]. Approaches such as time-restricted eating (TRE) and front-loading caloric intake earlier in the day show promise in improving metabolic outcomes and mitigating circadian disruption [27–32].

Importantly, adherence to the Mediterranean Diet (MD), characterized by high consumption of plant foods, moderate intake of fish, dairy, and eggs, and low intake of meat [33], appears to vary by chronotype. Studies in Spain and Italy consistently associate morning chronotypes with greater adherence to the MD, including higher intake of fruits, legumes, and olive oil, and lower consumption of meat [17, 34, 35]. Mechanistically, lower adherence to the MD among evening chronotypes may reflect the combined influence of social jet lag [23, 36] and personality-related factors, such as reduced conscientiousness and self-control [22].

The MD has consistently demonstrated protective effects against multiple metabolic outcomes in adults [37]. Evidence from more than 60 studies, involving over 1.1 million participants, shows significant inverse associations between MD adherence and type 2 diabetes (T2D), overweight, and obesity [37]. High adherence improves insulin sensitivity, modulates glycemic control, and reduces systemic inflammation and oxidative stress, as supported by observational and interventional evidence, including the *Prevención con Dieta Mediterránea* (PREDIMED) trial, which reported a 20% reduction in T2D incidence with the MD supplemented with extra virgin olive oil (EVOO) or nuts [38]. Moderate inverse associations with overweight and obesity likely reflect the MD’s high fiber content, low energy density, and low glycemic load, which modulate pathways relevant to weight regulation [37]. Findings for metabolic syndrome, dyslipidemia, and hyperuricemia are less consistent, though several longitudinal analyses indicate small but significant protective effects [37].

Overall, the MD constitutes a nutritionally balanced, culturally adaptable, and environmentally sustainable dietary pattern, characterized by minimally processed plant foods, healthy fats, seafood, and limited red and processed meat [39, 40]. Its incorporation into public health initiatives and clinical guidance is supported by robust evidence for T2D and obesity prevention, although structural barriers, such as food accessibility and socioeconomic disparities, remain critical considerations for widespread implementation [41–45]. Increasing evidence also indicates that chrononutrition and the MD exert independent yet complementary effects on metabolic health and sleep regulation. Beyond its established cardiometabolic benefits, the MD is consistently associated with improved sleep quality and duration [46, 47]. Observational studies further show that adherence to the MD varies by chronotype, with evening or intermediate types demonstrating lower adherence than morning types [47, 48], suggesting that chronotype shapes dietary behaviors and may influence the effectiveness of nutritional interventions.

On 16 November 2025, the MD will mark the fifteenth anniversary of its inclusion in the UNESCO Representative List of the Intangible Cultural Heritage of Humanity [49]. UNESCO emphasized that the MD extends beyond food choices, encompassing agricultural practices, culinary skills, and the convivial sharing of meals, with marketplaces serving as key sites of cultural transmission. Nonetheless, current epidemiological research, typically based on food-frequency questionnaires, has often overlooked qualitative aspects such as cooking methods, food combinations, conviviality, biodiversity, and production systems, focusing instead on quantitative assessment. This reductionist approach contrasts with recent revisions of the MD pyramid, which highlight seasonality, biodiversity, eco-friendly practices, and local traditions as essential components [50]. Accordingly, the MD should be conceptualized not merely as a dietary pattern but as a holistic and sustainable cultural model integrating nutritional, environmental, and social dimensions.

Taken together, these observations support the hypothesis that the integration of the MD principles into chrononutritional frameworks may offer synergistic benefits. On the one hand, the MD provides a high-quality dietary pattern recognized for its cardiometabolic and neuroprotective properties; on the other, chrononutrition contibutes the temporal alignment necessary to optimize metabolic and behavioral outcomes. Tailoring MD recommendations according to chronotype, for instance, by adapting meal timing, frequency, and macronutrient distribution to individual circadian preferences, may enhance adherence, improve sleep regulation, and amplify the protective effects of the diet. This conceptual fusion could form the basis of an updated model of nutritional practice, where both diet quality and circadian alignment are considered essential determinants of long-term health. The current representation of the food pyramid proposed by the *Italian Society of Human Nutrition* (SINU) [51], while serving as a valuable reference for promoting a healthy dietary pattern, does not incorporate key elements of chronobiology, such as individual chronotype and sleep hygiene, which are now recognized as critical determinants in defining a modern Mediterranean lifestyle.

In light of these considerations, this joint Consensus Statement of the *Italian Society of Endocrinology* (SIE) and the *Italian Association of Dietetics and Clinical Nutrition* (ADI) introduces a novel iconographic representation of the MD pyramid, reformulated according to a circadian-based approach. This integrated model seeks to promote a paradigm more consistent with current scientific knowledge, emphasizing the synchronization of dietary rhythms with individual circadian patterns as an essential component in the prevention and management of metabolic and cardiovascular diseases.

## Chrononutrition: Circadian Rhythm and Personalized Nutrition

Chrononutrition is a dietary approach focused on aligning eating habits with the human biological clock [24, 52]. It is based on the concept that metabolism fluctuates throughout the day, and that eating patterns that are not aligned with individual biological rhythms are associated with increased adiposity and cardiometabolic risk factors [24, 53, 54].

Chrononutrition therefore addresses three key dimensions of eating behavior: the timing of food intake, the frequency of meals, and the regularity of eating patterns [14, 25]. It represents an emerging field of research that investigates how circadian rhythms interact with meal timing and influence metabolic health.

To understand this approach, it is essential to consider the biology of circadian rhythms. The circadian rhythm (from the Latin *circa diem*, “about a day”) regulates the expression of clock-related genes in an approximately 24-hour cycle. Several physiological and metabolic functions, including hormone secretion, body temperature, feeding, and sleep behaviors, follow this periodicity and are adapted to daily cycles by endogenous circadian clocks [55]. The circadian system consists of two main components: the central (“master”) clock located in the suprachiasmatic nucleus (SCN) of the hypothalamus, and a network of peripheral clocks distributed throughout many tissues, such as the liver, gastrointestinal tract, pancreas, skeletal muscle, and adipose tissue [56].

The SCN receives light signals from the retina and uses the external dark-light cycle to orchestrate the peripheral clocks. These clocks are synchronized by powerful *Zeitgebers* (time-givers), which include the primary cue of light exposure, as well as key behavioral and environmental factors, such as the sleep-wake cycle, physical activity, and also dietary patterns [57, 58]. Peripheral clock synchronization is achieved not only through a “hierarchical” control of the SCN, but also through “horizontal” signals such as gastrointestinal and liver-derived metabolites, adipose tissue cytokines, and the timing of feeding [55]. Key mediators of this integrated network include the autonomic nervous system, cortisol, and melatonin, all of which play a central role in circadian homeostasis [59].

The circadian system is generally synchronized with the external light-dark cycle of ~ 24 h; however, interindividual variations in circadian timing can lead to distinct behavioral patterns.

These differences have been described as chronotypes and influence not only sleep-wake cycles, but also activities, eating behaviors, and metabolic outcomes [11, 24]. Chronotype refers to an individual trait that determines variability in circadian phase among people, reflecting when an individual is most alert and responsive to environmental cues. It is generally classified into “morning”, “evening” (commonly referred to as “larks” and “owls”), or “intermediate” categories, reflecting different preferences for daily activity and food intake patterns [11, 60].

Questionnaires are the most widely used method to assess chronotype, including the Morningness–Eveningness Questionnaire (MEQ) and its short version (rMEQ), the Composite Scale of Morningness (CSM), and the Munich Chronotype Questionnaire (MCTQ), which also enables the evaluation of “social jet lag” by comparing sleep timing on working day and work-free days [1, 11, 36] (Box 1).

**Box 1** Practical tools for chronotype assessment in clinical practice

**Table:** 

**Why this matters:** Although chronotype is conceptually addressed, simple and rapid tools can help clinicians apply chronotype-informed MD recommendations. **Quick clinical screening (≤ 30 s)**: • “At what time of day do you feel most alert and productive?” • “If you could freely choose your schedule, what time would you naturally wake up and go to bed?” • “Do you usually feel hungry in the morning, or do you prefer to eat later in the day?” **Validated questionnaires**: • MEQ (19 items) or rMEQ (5 items) – Morningness–Eveningness Questionnaire: quick and widely used in clinical and research settings. • CSM – Composite Scale of Morningness (13 items): offers a more detailed chronotype classification. • MCTQ – Munich Chronotype Questionnaire (19 items): useful to assess “social jetlag” and irregular sleep–wake patterns. Implementation tips: • Incorporate the rMEQ into standard intake forms. • Use the MCTQ in individuals reporting irregular schedules, late eating, or sleep disturbances. • Combine chronotype classification with usual meal timing (first/last meal) to identify circadian misalignment.	

Dim light melatonin onset can also be used, and it is considered the most reliable measure to assess individual’s chronotype, although its application is less feasible in diverse settings [61].

Morning chronotypes tend to schedule activities and food intake early in the day, whereas evening chronotypes generally delay them toward late afternoon or night. Intermediate chronotypes represent a midpoint between these patterns, showing a more adaptable distribution of daily behaviors and metabolic processes [24].

Evening chronotype has been associated with metabolic disorders [18]. Individuals with this chronotype are more likely to skip meals (particularly breakfast), show lower consumption of fruits and vegetables, and rely more on energy-dense foods and stimulants [14]. These behaviors may contribute to the increased incidence of obesity, metabolic dysfunction, T2D, gastrointestinal disorders, psychiatric symptoms, and cardiovascular risk factors observed in evening chronotypes compared with morning chronotypes [18, 19, 62–64]. Moreover, evening chronotype has been reported to co-occur with disordered eating and eating disorders [65, 66].

The mismatch between the intrinsic circadian clock and the external environment (e.g., due to shift work) leads to a state of chronodisruption, defined as the breakdown of correct phasing between internal biological systems and the external *Zeitgebers* [67]. This desynchronization involves an uncoupling of the central SCN clock from peripheral clocks and is particularly evident with changes in the activity-rest or feeding-fasting cycles.

In addition to biological and interindividual variability, lifestyle and contextual factors (such as sedentariness, chronic stress, screen exposure) further aggravate circadian disruption and contribute to adverse health outcomes [68–70]. In this context, particular attention has been given to quality and quantity of sleep: short sleep duration is consistently associated with an increased risk of obesity and other chronic disorders [71]. These associations may be due to alterations in hormones involved in energy balance, together with extended opportunities for food intake, and fatigue-related reductions in daily physical activity [72, 73].

To counteract these effects, nutritional strategies that restrict food intake to defined daily windows have been associated with potential benefits for metabolic health [55]. Recent evidence suggests that such approaches may mitigate features of metabolic disorders [28, 29, 74]. Various chrononutritional approaches have been described, that include several patterns: chronic energy restriction lowers total daily intake while maintaining meal frequency, whereas intermittent fasting alternates periods of fasting with hours of unrestricted food intake [75]. Within this framework, TRE represents a specific form of chrononutrition, where the eating window is consistently reduced (from 4 to 12 h per day), without imposing caloric restriction [30, 76]. Although chrononutrition and meal timing strategies show potential benefits, current evidence is largely based on short-term interventions or small sample studies, while robust long-term clinical trial data are still lacking. As a result, the extent to which these approaches can replace or complement calorie restriction for obesity management remains uncertain [57, 77].

### Chrononutrition: the Timing of Nutrients

Recent advances have highlighted the importance not only of what we eat, but also when we eat. Aligning nutrient intake with these biological rhythms may help prevent metabolic disorders such as obesity, T2D, and metabolic syndrome.

Emerging evidence suggests that the timing of protein ingestion represents a crucial determinant in maintaining skeletal muscle homeostasis across the circadian cycle. During nocturnal fasting, muscle protein synthesis rates decline and proteolysis prevails, resulting in a net negative protein balance. Strategic protein intake aligned with circadian rhythms, particularly pre-sleep protein ingestion, has emerged as a promising intervention to mitigate this catabolic window.

Experimental data have consistently demonstrated that protein consumed before sleep is effectively digested and absorbed throughout the night, leading to a sustained elevation in plasma amino acid availability and stimulation of overnight muscle protein synthesis [78, 79]. The ingestion of 40 g of protein prior to sleep has been shown to increase myofibrillar protein synthesis rates by approximately 33% compared with placebo, thereby shifting the whole-body protein balance from neutral to positive during sleep [78]. In contrast, lower doses (e.g. 20 g), even when fortified with leucine, fail to elicit comparable anabolic responses, likely due to insufficient amino acid precursor availability during the extended postprandial period [78]. These findings indicate that higher protein doses are required during the prolonged overnight fast, to sustain adequate aminoacidemia and stimulate *de novo* muscle protein accretion.

From a chronic perspective, regular pre-sleep protein consumption (20–40 g casein, ~ 30 min before bedtime) has been associated with greater adaptive responses to resistance exercise, such as increases in muscle fibre cross-sectional area, strength, and lean mass in young adults, albeit with more modest outcomes in older populations [79]. Nonetheless, despite these encouraging results, available data are still limited and partly confounded by differences in total protein intake across studies, making it difficult to isolate a distinct “timing effect” [79]. Moreover, the temporal kinetics of amino acid availability suggest that muscle protein synthesis stimulation may occur predominantly within the first hours following ingestion, after which muscle sensitivity to amino acids wanes, despite sustained elevations in plasma aminoacidemia [80].

Mechanistically, these anabolic effects may be partly mediated through interactions with the growth hormone (GH)–insulin-like growth factor-I (IGF-I) axis. GH secretion displays a strong circadian rhythm, with its major pulse occurring shortly after sleep onset. Nutritional status exerts profound modulatory effects on this axis: fasting or caloric restriction enhances GH pulsatility, whereas prolonged nutrient deprivation leads to peripheral GH resistance, characterised by elevated GH levels and suppressed IGF-I synthesis [81]. Amino acids act as potent regulators of somatotroph function; basic amino acids such as arginine, lysine, and histidine robustly stimulate GH release by inhibiting hypothalamic somatostatin output, while branched-chain amino acids, particularly leucine, modulate both GH and IGF-I secretion, albeit with variable potency depending on dose, age, and metabolic status [81]. Furthermore, high-protein diets and dairy-based proteins have been associated with increased basal GH and circulating IGF-I concentrations [81], reinforcing the bidirectional link between amino acid availability and somatotropic activity.

Collectively, these data support the hypothesis that pre-sleep protein ingestion may potentiate the nocturnal anabolic milieu by synchronising amino acid availability with the endogenous GH surge, thereby enhancing the efficiency of protein synthesis and attenuating overnight muscle protein degradation. This nutrient timing approach could represent a physiologically grounded strategy to optimise muscle mass maintenance, particularly in populations prone to anabolic resistance or accelerated muscle loss, such as older adults or individuals under caloric restriction.

Epidemiological evidence indicates that carbohydrate consumption earlier in the day may confer protective effects against the onset of metabolic syndrome and T2D. One longitudinal study involving 1,488 individuals aged 43–53 years, followed over a 10-year period, demonstrated that replacing 5% of dietary fat with carbohydrates at breakfast was associated with a reduced risk of developing metabolic syndrome, as well as lower levels of circulating triglycerides and less visceral adiposity [10]. Conversely, excessive evening carbohydrate intake has been linked to elevated morning blood glucose levels. Several investigations have explored the influence of meal timing and glycemic index (GI) on postprandial insulin and glucose responses. In a randomized crossover trial, consumption of a high-GI meal in the evening elicited greater glycemic and insulinemic responses compared to the same meal consumed in the morning [82].

These findings are consistent with the known circadian modulation of glucose metabolism, whereby insulin sensitivity peaks during daytime and significantly diminishes in the evening due to circadian fluctuations in insulin secretion and action [83]. Both insulin and cortisol, key hormones in glucose homeostasis, exhibit circadian oscillations that contribute to time-of-day-dependent variations in metabolic responses [84]. Therefore, disruptions in meal timing or diet composition— central concepts of chrononutrition—may alter circadian rhythms and be implicated in the pathogenesis of metabolic disorders, including T2D.

Furthermore, breakfast composition also appears to modulate metabolic responses to subsequent meals. High carbohydrate intake at breakfast has been shown to regulate circadian clocks and enhance glucose and insulin metabolism, and has also been associated with lower fat and alcohol consumption throughout the day [85]. This dietary pattern correlates with reduced risk of developing T2D. At night, despite an increase in insulin secretion, elevated insulin clearance results in lower insulin sensitivity and glucose tolerance, further substantiating the detrimental metabolic effects of evening carbohydrate intake [85].

In a randomized crossover trial, Maki et al. compared a high-protein breakfast (two eggs per day, 6 days *per* week) with an isocaloric, carbohydrate-rich control breakfast for four weeks. The egg-based breakfast led to a smaller reduction in LDL-cholesterol but was associated with decreased systolic blood pressure and did not adversely impact insulin sensitivity or carbohydrate metabolism compared to the control condition. Although high-protein breakfast was linked to a modest increase in energy intake from non-study foods, this did not result in weight gain [86].

Emerging in vitro evidence suggests that certain fatty acids, such as monounsaturated (MUFAs) and polyunsaturated fatty acids (PUFAs), may exert epigenetic effects, particularly on genes involved in circadian regulation. Specifically, MUFAs and PUFAs intake modulate methylation levels of CpG sites in the promoter region of the CLOCK gene. MUFAs intake, including from olive oil, is inversely associated with CLOCK methylation, while PUFAs have a positive association [87]. These epigenetic modifications may influence circadian genes expression and thus serve as potential biomarkers for weight loss.

Genetic variability also interacts with dietary timing. For instance, individuals carrying the C allele of the CLOCK 3111T/C polymorphism (rs1801260) demonstrate a more stable circadian rhythm than TT homozygotes, and respond more favorably to diets high in MUFAs (> 13% of total energy intake), whereas diets high in saturated fatty acids (SFAs) are associated with increased visceral adiposity [87].

A study conducted in Spain between 2006 and 2011 assessed chronotype using the MEQ in bariatric surgery patients. Results indicated that evening chronotypes were more likely to exhibit severe obesity and showed poorer postoperative weight loss. Additionally, chronotype interacted with the CLOCK 3111T/C polymorphism, further influencing obesity outcomes [88].

Another gene of interest is PLIN1, which encodes perilipin, a protein regulating triglyceride storage in adipocytes and playing a central role in lipid droplet formation and catecholamine-induced lipolysis. PLIN1 expression is under circadian control and influences energy balance and adiposity regulation [89]. In the ONTIME study involving 1,287 overweight and obese participants, genotyping of PLIN1 single nucleotide polymorphisms revealed that individuals carrying the AA genotype of PLIN1 114,995 A/T who consumed lunch later in the day (after 15:00) experienced significantly less weight loss compared to early eaters, highlighting the gene-diet interaction in weight management [90].

Recent findings further support the role of chrononutrition in women’s health [91]. A 2025 cross-sectional study of 100 postmenopausal women with overweight or obesity found that a higher evening lipid intake was significantly associated with increased menopausal symptoms, particularly heart discomfort. Conversely, higher morning lipid consumption was linked to fewer symptoms. These associations remained significant after adjusting for confounding variables, highlighting that not only nutrient quality but also timing, especially of lipid intake, can influence cardiometabolic and menopausal outcomes in this population [91].

### Mediterranean Diet and Diseases: Current Scientific Evidence

The MD is characterised by a high consumption of plant-based foods, such as vegetables, fresh fruits, nuts, legumes, and cereals, preferably whole grains—along with olive oil as the main source of monounsaturated fats [92]. It also includes moderate intake of fish, seafood, eggs, poultry, milk, and dairy products, with limited consumption of red meat, sweets, and processed meats [92], reflecting a preference for local, seasonal, and minimally processed ingredients [93]. Historically associated with greater longevity and lower chronic disease burden in Mediterranean populations [94], the MD is now supported by extensive epidemiological and clinical evidence demonstrating its effectiveness in preventing and managing non-communicable diseases, such as cardiovascular disease, T2D, metabolic syndrome, and obesity (Table 1).

**Table 1:** Key studies on Mediterranean Diet and diseases

Design/Population	Main Findings	Ref.
*Seven Countries Study* – 15 cohorts, 11,579 healthy men aged ≥ 40 years, 15-year follow-up	Mortality increased with higher saturated fat intake and was inversely associated with MUFAs intake. No associations with PUFAs, protein, carbohydrates, or alcohol. Higher MUFAs/SFAs ratio predicted lower all-cause mortality. Combined with age, BP, cholesterol and smoking it explained 85% of all-cause mortality variance (96% CHD, 55% cancer, 66% stroke). Lowest mortality in cohorts with high olive oil intake.	[95]
PREDIMED cohort analysis − (7216 adults; RCT arms prospectively studied; 4.8y median follow-up)	Higher total olive oil and EVOO intake reduced cardiovascular disease risk by 35% (HR 0.65) and 39% (HR 0.61) vs. lowest tertile. Higher total olive oil intake reduced cardiovascular mortality by 48% (HR 0.52). Each + 10 g/day EVOO lowered CVD risk by 10% and cardiovascular mortality by 7%. No association with all-cause mortality or cancer. Benefits were more evident in MD intervention groups.	[96]
Systematic review − (57 cohort/retrospective studies; >1.8 M participants; mean follow-up 13 years)	Greater adherence to the MD was significantly associated with reduced all-cause mortality (RR 0.96 per 1-point increase in adherence score). Based on 346,034 deaths (~ 19% of participants). Strong evidence for enhanced longevity.	[93]
Systematic review & meta-analysis − (60 studies; >1.1 M participants)	High adherence to the MD reduced risk of T2D (RR 0.96), overweight (OR 0.94), obesity (OR 0.95), metabolic syndrome (RR 0.98) and hyperuricemia (OR 0.43). Effects on hypercholesterolemia and hypertriglyceridemia were inconsistent. Evidence quality moderate-to-high.	[37]

SFAs, saturated fatty acids; MUFAs, monounsaturated fatty acids; PUFAs, polyunsaturated fatty acids; CHD, coronary heart disease; BP, blood pressure; EVOO, extra-virgin olive oil; MD, Mediterranean Diet; CVD, cardiovascular disease; HR, hazard ratio; RCT, randomized controlled trial; RR, relative risk; OR, odds ratio; T2D, type 2 diabetes.

The first robust evidence in this field came from the *Seven Countries Study*, which evaluated 15 cohorts comprising 11,579 healthy men aged ≥ 40 years, with 2,288 deaths recorded over 15 years of follow-up [95]. Mortality was positively associated with the proportion of energy from SFAs, inversely associated with MUFAs, and unrelated to PUFAs, proteins, carbohydrates, or alcohol. All-cause mortality was inversely related to MUFAs-to-SFAs ratio, which, together with age, blood pressure, serum cholesterol, and smoking, explained 85% of the variance in all-cause mortality, 96% in coronary heart disease, 55% in cancer, and 66% in stroke mortality. Oleic acid accounted for almost all inter-cohort differences in MUFAs intake, and mortality rates were lowest in cohorts relying on olive oil as the main fat source [95].

A recent meta-analysis including 60 studies and more than 1.1 million participants confirmed that higher adherence to the MD was consistently associated with reduced risk of T2D (RR, 0.96; 95% CI, 0.95–0.97), overweight (OR, 0.94; 95% CI, 0.91–0.97), and obesity (OR, 0.95; 95% CI, 0.93–0.97), with protective effects also observed for metabolic syndrome (RR, 0.98; 95% CI, 0.98–0.99) and hyperuricemia (OR, 0.43; 95% CI, 0.25–0.75). Associations with hypercholesterolemia and hypertriglyceridemia were inconsistent and inconclusive [37].

According to the *National Guidelines* published by the *Italian National Institute of Health*, a systematic review including 57 cohort and retrospective studies (1,833,267 participants; follow-up range, 2–60 years; mean, 13 years) investigated the association between the MD adherence and all-cause mortality. During the observation period, 346,034 deaths were recorded, representing approximately 19% of the total population. Pooled analysis revealed a statistically significant inverse association between adherence to the MD and mortality risk (RR, 0.96; 95% CI, 0.95–0.97 for each one-point increase in adherence score), indicating a protective effect of greater adherence to the MD against all-cause mortality [37].

Among the most influential studies is the PREDIMED trial, which assessed the relationship between EVOO consumption and cardiovascular outcomes in individuals at high cardiovascular risk. A total of 7,216 men and women aged 55–80 years from this multicentre, randomized, controlled trial were assigned to a MD supplemented with nuts, a MD supplemented with EVOO, or a low-fat control diet, and followed for a median of 4.8 years [96]. Cardiovascular events and mortality were verified through medical records and registries, and olive oil intake measured by validated food-frequency questionnaires. During follow-up, 277 cardiovascular events and 323 deaths occurred. Participants in the highest tertile of baseline total olive oil and EVOO intake had 35% (HR, 0.65; 95% CI, 0.47–0.89) and 39% (HR, 0.61; 95% CI, 0.44–0.85) lower cardiovascular risk, respectively, compared with the lowest tertile. Higher baseline total olive oil intake was also associated with a 48% reduction in cardiovascular mortality (HR, 0.52; 95% CI, 0.29–0.93). Each 10-g/day increase in EVOO reduced cardiovascular disease and mortality risk by 10% and 7%, respectively. No significant associations were found for cancer or all-cause mortality. The associations between cardiovascular events and EVOO intake were significant in the MD intervention groups but not in the control group.

Collectively, these findings confirm that higher olive oil consumption—particularly EVOO—is associated with reduced cardiovascular disease and mortality risk in individuals at elevated cardiovascular risk [96]. This aligns with recommendations from the *Italian National Institute of Health*, which endorses the adoption of MD enriched with EVOO over a low-fat diet for reducing T2D incidence and improving cardiometabolic health [37].

## Mediterranean Diet Based on Chrononutrition and Chronotype

Current evidence indicates that diet and sleep quality are two interconnected dimensions with a profound impact on human health [97, 98]. However, recent decades have witnessed shifts in dietary behaviors and lifestyle from traditional Mediterranean patterns, contributing to the spread of disruptive habits [99]. Historical accounts by Ancel Keys about southern Italian farmers, together with more recent descriptions of older residents in Mediterranean islands, depict a lifestyle marked by early waking, a modest midday meal often followed by a brief rest, and an early evening meal constituting the main caloric intake [100]. These routines suggest that the traditional Mediterranean lifestyle was naturally aligned with circadian rhythms.

Emerging evidence supports this link: individuals with a morning chronotype, defined by an innate preference for early wake and activity onset in the morning, display greater adherence to the MD [48]. This observation underlines the importance of synchronizing dietary patterns with biological rhythms, a core principle of chrononutrition. In this context, lifestyle features such as consistent sleep schedules, daylight-aligned activity, occasional mid-day rest, and reduced eating occasions represent distinctive traits of the traditional Mediterranean lifestyle, markedly different from contemporary living patterns [101]. From an evolutionary and circadian perspective, humans appear better adapted to consuming food during restricted daytime periods [102].

Such temporal eating patterns have been shown to modulate metabolic and neuronal network activity, thereby influencing a wide range of physiological processes [103]. They have also been linked to reduced risk of non-communicable diseases, including cardiovascular conditions [104], metabolic risk factors [105], certain cancer types with implications for prevention and survival [106], as well as affective and cognitive disorders [107]. Within this framework, research has begun to examine the interplay between the MD and TRE strategies, such as limiting food intake to specific daily windows or fasting during Ramadan. These patterns, when embedded in a Mediterranean dietary context, have been associated with improvements in metabolic health [108, 109].

Nevertheless, recent randomized controlled evidence indicates that the benefits of TRE may not necessarily exceed those of conventional Mediterranean-style energy-restricted diets. For instance, an early time-restricted carbohydrate (eTRC) intervention in individuals with T2D yielded similar improvements in glycemic control, body weight, and cardiometabolic markers comparable to a Mediterranean-style control diet [110]. These findings suggest that while TRE is a feasible and effective strategy, its advantages over traditional MD approaches remain uncertain.

By contrast, chronodisruption and the evening chronotype, most commonly reflected by social jetlag (misalignment between internal biological rhythms and externally imposed social schedules), have been associated not only with poorer sleep quality but also with less healthy dietary behaviors. In particular, individuals experiencing social jetlag tend to consume meals later in the day, show irregular eating patterns, and exhibit reduced adherence to the MD [111, 112]. These disruptions may contribute to weight gain, metabolic alterations, and a decline in overall diet quality, underscoring how circadian misalignment can counteract the potential benefits of a Mediterranean lifestyle.

Despite these compelling associations, it is important to acknowledge that chronotype research is still limited by its reliance on observational designs, heterogeneous assessment tools, and short-term or small clinical trials; as a result, causal pathways and the long-term clinical impact of aligning MD patterns with chronobiology remain to be fully established.

### Metabolic Flexibility in Health and Disease

Metabolic flexibility refers to the capacity of organisms (or tissues) to adapt fuel oxidation to nutrient availability and energy demand, for example, efficiently switching from lipid oxidation during fasting to glucose oxidation in the postprandial state, and vice versa [113]. In healthy conditions, this adaptability prevents excessive postprandial glucose level and ensures energy supply during fasting. It also supports healthy body composition and a proper adipose tissue function, promoting lipid storage and mobilization while limiting ectopic fat accumulation [114, 115].

At the cellular level, metabolic flexibility relies on mitochondria, which integrate substrate flux, redox balance, oxidative capacity, and quality control processes such as fusion, fission, and mitophagy. It also depends on coordinated signaling and transcriptional regulation involving pathways such as AMP-activated protein kinase (AMPK), sirtuins (SIRT), peroxisome proliferator-activated receptor gamma coactivator-1 alpha (PGC-1α), and the mechanistic target of rapamycin (mTOR) [113, 116]. Importantly, impaired mitochondrial function or dysregulated mitochondrial dynamics alter the organelle’s ability to adapt to changing fuel loads, thereby contributing to metabolic inflexibility [117].

Clinically, metabolic inflexibility is frequently observed in conditions such as insulin resistance, obesity, T2D, and metabolic dysfunction-associated fatty liver disease, and may represent an early defect in the pathogenesis of metabolic disease [118, 119]. This impaired metabolic adaptability is also evident in sarcopenia [120] and neurodegenerative processes, leading to increased neuroinflammation and accelerated cognitive decline [121].

Excessive or unbalanced nutrient intake, especially when combined with diets rich in ultra-processed foods, physical inactivity, and circadian disruption may impair nutrient sensing, promote mitochondrial dysfunction, and alter metabolic homeostasis [122]. This contributes to a state of metabolic inflexibility, described as “mitochondrial indecision” leading to metabolic gridlock [117].

In metabolically healthy individuals, substrate oxidation shifts in response to nutrient availability and circadian cues: a carbohydrate meal increases insulin and respiratory quotient, reflecting a switch from lipid to glucose oxidation. During the post-absorptive or fasting state, fat oxidation predominates and the respiratory quotient declines [119]. These transitions are regulated not only by metabolic status but also by meal timing, aligning with the body’s internal clock to optimize energy utilization [123].

**Fig. 1:**
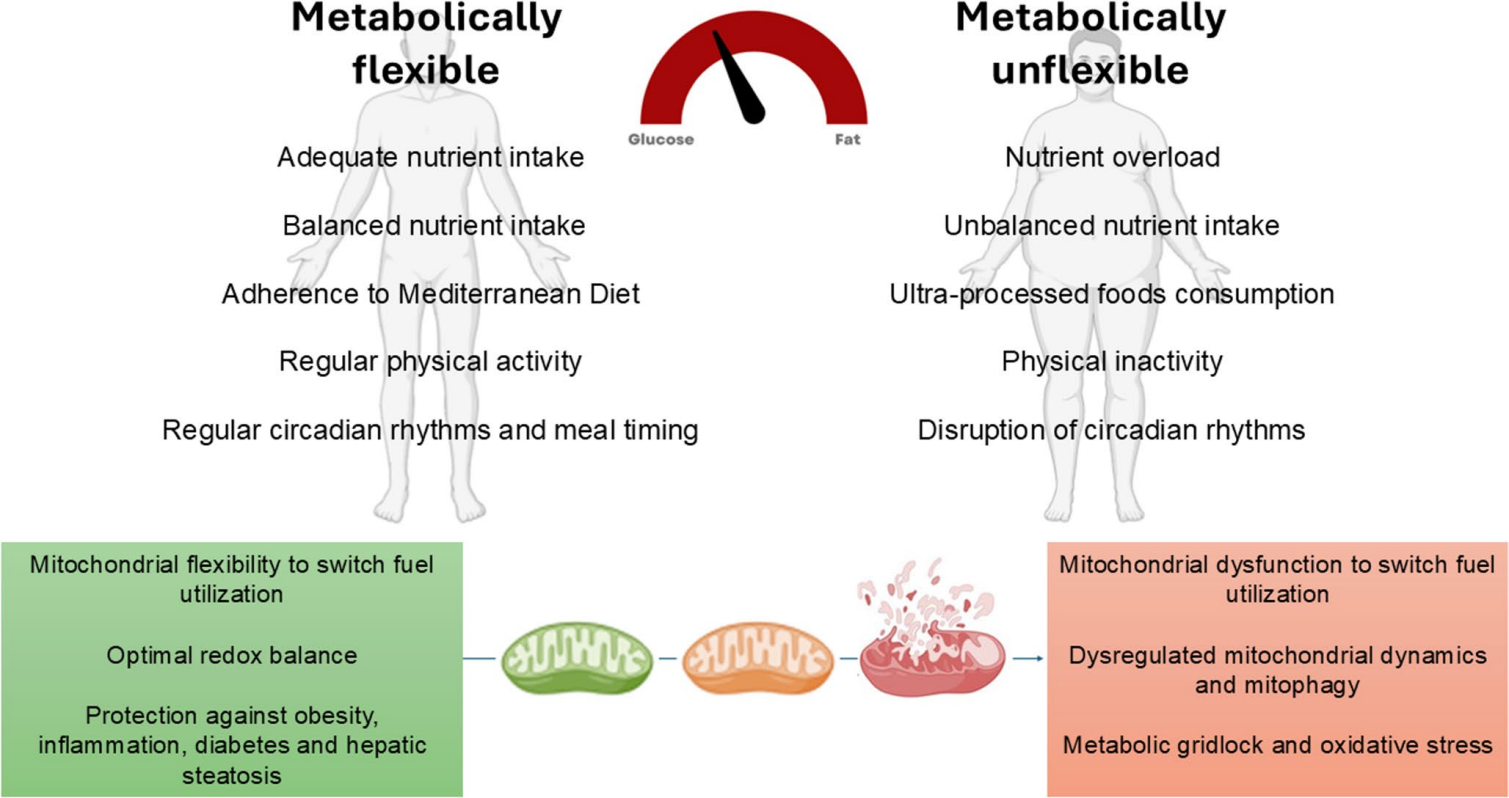
Overview of metabolic flexibility versus metabolic inflexibility and their mitochondrial and clinical consequences. Metabolically flexible individuals maintain a balanced nutrient intake, follow a Mediterranean Diet, engage in regular physical activity, and keep circadian rhythms and meal timing aligned. Their mitochondria efficiently shift between fuel, sustain an optimal redox balance, and exhibit preserved mitochondrial dynamics, mitophagy, and enhanced mitochondrial biogenesis. Together, these processes support protection against obesity, inflammation, diabetes, and hepatic steatosis. In contrast, metabolically inflexible individuals are characterised by nutritional overload, unbalanced diets rich in ultra-processed foods, physical inactivity, and circadian rhythm disruption. These conditions lead to impaired mitochondrial fuel switching, reduced mitochondrial biogenesis, dysregulated mitochondrial dynamics and mitophagy, metabolic stalling, and elevated oxidative stress

By contrast, metabolic inflexibility, commonly observed in obesity, is characterized by impaired fuel switching, persistent mixed-substrate oxidation, and mitochondrial overload [119, 124, 125]. Emerging evidence highlights that delayed eating times, such as eating late at night or during the biological rest phase, exacerbate this dysfunction by desynchronizing central and peripheral metabolic pathways. Over time, these patterns contribute significantly to the development of non-communicable diseases [75]. An overview of metabolic flexibility versus metabolic inflexibility and their mitochondrial and clinical consequences is shown in Fig. 1.

### Chrononutrition and Metabolic Flexibility

Chrononutrition explores how meal timing affects metabolism through the body’s circadian clocks [32, 126]. Organs like liver and muscle have their own clocks, and eating acts as a key timing signal. Aligning meals with these clocks improves insulin sensitivity, nutrient use, and energy balance [127–130].

In the context of chronotype, individuals whose circadian phase is shifted (e.g. evening chronotypes) may experience a misalignment between habitual eating times and their metabolic optimum, which could compromise metabolic flexibility. For example, eating late at night, particularly carbohydrates-meal, (when endogenous insulin sensitivity is lower) forces mitochondria to process nutrients at a non-optimal time, increasing residual substrate burden, oxidative stress, and possibly promoting inflexibility [131, 132].

Preclinical evidence suggests that mistimed feeding (during the inactive phase) abolishes daily rhythms in mitochondrial respiration and disrupts the expression of genes related to mitochondrial biogenesis and fission/fusion machinery [133]. Thus, appropriate temporal coordination between feeding windows and circadian metabolic readiness is likely essential to preserve mitochondrial plasticity and systemic metabolic flexibility.

### Bioactive Components of the Mediterranean Diet and Metabolic Flexibility

The MD is rich in bioactive compounds (polyphenols, omega-3 fatty acids, fiber, phenolic acids, etc.) which can modulate mitochondrial function, biogenesis, redox balance, and signal transduction, thereby enhancing MF [134]. Several key mechanisms underlie this beneficial effect.

A central pathway involves the activation of the AMPK/SIRT/PGC-1α axis. Polyphenols such as resveratrol, along with omega-3 fatty acids, oleic acid, and conjugated linoleic acids, can activate AMPK and elevate intracellular nicotinamide adenine dinucleotide (NAD⁺) levels [135–138]. This, in turn, engages SIRT1 and SIRT3, promoting mitochondrial biogenesis, enhancing fatty acid oxidation, strengthening antioxidant defences, and improving overall mitochondrial quality [139, 140]. This signaling cascade is crucial for enabling efficient substrate switching in response to energy demands, a hallmark of metabolic flexibility [141–143].

Another important mechanism relates to the regulation of mitochondrial dynamics and mitophagy. Bioactive compounds found in the MD have been shown to promote mitochondrial fusion and support the removal of dysfunctional mitochondria through mitophagy, thereby preserving mitochondrial integrity [144]. Hydroxytyrosol, a potent phenolic compound in EVOO, has demonstrated the ability to restore expression of electron transport chain complexes and stimulate mitochondrial biogenesis genes, such as nuclear respiratory factor 1 (NRF1) and mitochondrial transcription factor A (TFAM), *via* AMPK-related pathways in preclinical models [145]. The MD also provides antioxidant and anti-inflammatory protection at the mitochondrial level. By reducing the production of mitochondrial reactive oxygen species (mtROS) and limiting oxidative stress, its bioactive components help preserve the redox environment necessary for efficient energy metabolism. Carotenoids like lycopene, and polyphenols like resveratrol, modulate key mitochondrial enzymes, help maintain membrane potential, and enhance respiratory efficiency, ultimately contributing to preserved mitochondrial function under metabolic stress [136–138].

Furthermore, short-chain fatty acids (SCFAs), particularly butyrate, produced through the fermentation of dietary fiber by the gut microbiota, have been shown to enhance mitochondrial capacity, improve glucose homeostasis, and reduce insulin resistance, as demonstrated in preclinical models of diet-induced metabolic dysfunction [146–150].

The composition of mitochondrial membranes is another target modulated by the MD components. Omega-3 fatty acids, including docosahexaenoic acid and eicosapentaenoic acid, and conjugated linoleic acid are incorporated into mitochondrial membranes, improving their fluidity and the efficiency of the electron transport chain, while also reducing ROS. These structural changes enhance the mitochondria’s ability to adjust substrate use in response to energy demands [138, 143].

Importantly, the synergistic effect of the overall dietary pattern amplifies these benefits. Rather than acting in isolation, the combination of polyphenols, omega-3 fatty acids, dietary fiber (and its downstream SCFAs), vitamins, and minerals contributes collectively to mitochondrial health, substrate adaptability, and metabolic resilience. The whole-diet approach of the MD promotes efficient cellular energy metabolism, particularly under conditions of stress or shifting energy demands.

### Mediterranean Diet Beyond Diseases: Economic Impact and Sustainability

In 2010, the Food and Agriculture Organization (FAO) and Bioversity International convened an international scientific symposium on “biodiversity and sustainable diets,” which resulted in a consensus definition of sustainable diets. According to this definition, sustainable diets are those with low environmental impact that support food and nutrition security and promote healthy lives for present and future generations. They are protective and respectful of biodiversity and ecosystems, culturally acceptable, accessible, economically fair and affordable, nutritionally adequate, safe, and healthy, while optimizing both natural and human resources [151].

The FAO projects that by 2050, global food production will need to increase by at least 60% to meet the demands of a larger and wealthier population with a growing appetite for animal-derived foods [152]. Achieving this target poses a substantial challenge for both food security and environmental sustainability, as natural resources are already under significant pressure and degradation, further exacerbated by the effects of climate change. At present, a key global priority is to safeguard natural resources for future generations while ensuring the availability of sufficient, nutritious, and safe food to meet the dietary needs of an expanding world population [153].

However, beyond its nutritional advantages, the MD also represents a sustainable dietary model that aligns with global environmental and climate objectives [154]. In the context of growing concerns regarding food security, climate change, and environmental pollution, adopting and preserving the MD can play a crucial role in promoting both human and planetary health. The MD is characterized by a high consumption of fruits, vegetables, whole grains, legumes, nuts, olives, and olive oil; moderate consumption of fish and poultry; and low consumption of red meat [155]. The MD is often promoted as a healthy and environmentally sustainable eating pattern that is socially and culturally acceptable and confers positive local economic impacts [156].

The traditional Mediterranean “lifestyle” expands this concept beyond diet to encompass adequate rest, regular physical activity, frugality, dietary diversity, and social engagement—such as conviviality through shared meal preparation and communal eating. It also prioritizes local, seasonal, ecological, and minimally processed foods that support biodiversity. According to the *National Guidelines* of the *Italian National Institute of Health*, the environmental sustainability of the MD is considered variable; however, high adherence is viewed as a sustainable choice in terms of environmental and seasonal resource use, and generally outperforms most alternative dietary patterns, except those characterized by markedly lower intakes of animal-derived products, such as vegan diets [37]. Greater adherence to the MD has been shown to be more sustainable than low adherence in 7 of 9 studies [157–162]. The MD was less sustainable than the vegan diet [163] but more sustainable than the current Italian diet in all three comparative analyses [164–166]. It was also consistently more sustainable than the Western diet in all the five studies analyzed [167–170]. However, it did not surpass vegetarian or EAT-Lancet dietary models in terms of overall sustainability [171, 172].

Moreover, the environmental sustainability of the MD is also rooted in the use of seasonal and locally sourced foods, commonly referred to as “zero-kilometres” (Km 0) products. This approach reduces both the economic and environmental costs associated with food importation, and lowers the overall energy footprint. Moreover, locally grown seasonal foods tend to be richer in micronutrients, as they are allowed to ripen naturally according to their proper seasonal cycle [156].

### The Fifteenth Anniversary as a UNESCO World Cultural Heritage

On the fifteenth anniversary of its recognition by UNESCO as an Intangible Cultural Heritage of Humanity (16 November 2010) [49], the MD should be reconsidered in its full complexity, as a living cultural system integrating nutritional, social, environmental, and spiritual dimensions rather than a mere list of foods. When inscribed on the Representative List, UNESCO described it as a constellation of skills, knowledge, practices, and traditions encompassing crop cultivation, harvesting, fishing, animal rearing, food processing and preservation, culinary techniques, and the convivial sharing of meals [173]. These practices, transmitted across generations, represent a key marker of identity for Mediterranean communities [174, 175], with marketplaces, home kitchens, and shared tables functioning as spaces where cultural memory and collective belonging are continuously reinforced [173, 176].

Despite this multidimensional definition, scientific literature has often reduced the MD to a quantitative dietary pattern assessed through consumption-frequency scores. While such operationalisations have generated important epidemiological evidence, they risk overlooking qualitative and contextual elements, such as culinary methods, food combinations, degree of processing, and the local and seasonal origin of ingredients—which critically influence health outcomes and sustainability [173]. For this reason, the updated MD pyramid have reintroduced principles including seasonality, biodiversity, cultural traditions, and environmentally responsible production, aligning the model with broader sustainability frameworks [50, 51].

Conviviality also represents a defining component of the Mediterranean tradition: shared meals support social bonds and psychological well-being [176]. Indices incorporating lifestyle and social components, such as the Mediterranean Lifestyle (MEDLIFE) index, are associated with significant reductions in overall and cancer-related mortality [177]. Meta-analyses similarly show lower risk of depression and cognitive decline among individuals with higher adherence to the Mediterranean pattern [178], potentially mediated by the psychosocial benefits of family meals, intergenerational exchange, and the sense of belonging fostered by communal dining [179]. Thus, conviviality functions as both a cultural feature and a determinant of health [176].

From an ecological perspective, the MD has been progressively framed as a paradigm of low environmental impact since the 1990 s [153]. International frameworks from FAO and CIHEAM endorse its multidimensional sustainability, emphasizing nutritional adequacy, biodiversity preservation, local economic support, and resource efficiency [151, 180, 181]. Empirical modelling consistently demonstrates that adherence to the MD reduces greenhouse gas emissions, land use, and eutrophication potential compared to Westernized diets [182], largely due to the predominance of plant-based foods, limited red meat intake, and the environmental value of legumes and permanent crops such as olive groves and fruit trees [183, 184]. These advantages are reported in national modelling scenarios, such as those in Spain, and in large-scale interventions like PREDIMED-Plus, where participants’ dietary choices were associated with markedly lower environmental footprints [185, 186].

Furthermore, religious and spiritual traditions across Judaism, Christianity, and Islam have historically shaped Mediterranean food cultures by defining norms, prohibitions, and ritual practices that influence dietary choices and timing [187]. Kosher and Halal rules [188], Lent fasting [188, 189], Ramadan fasting, and other ritual abstinences [189, 190] act not only as theological prescriptions but as cultural practices that reinforce community cohesion, continuity, and the symbolic dimension of eating [187]. Although the metabolic effects of fasting regimes vary, their broader significance lies in the social and spiritual meanings they confer.

Taken together, these aspects indicate that, fifteen years after UNESCO recognition, the MD must be approached as a multidimensional construct. Its value lies not only in its established health benefits but also in its expression of conviviality, its environmentally sustainable model of production and consumption, and its deep embedding in the spiritual and symbolic life of Mediterranean societies. The challenge for scholars, clinicians, and policymakers is to move beyond narrow, frequency-based definitions and adopt integrative tools that capture the full spectrum of Mediterranean dietary heritage, from biodiversity and seasonality to culinary skills, convivial practices, and religious foodways, so that the MD can continue to support well-being, social cohesion, and ecological resilience.

### A Novel Mediterranean Diet Pyramid Tailored To Chronotype and Lifestyle Factors

Despite robust evidence supporting the role of circadian biology and meal timing in metabolic regulation, current nutritional recommendations do not consider chronotype-specific differences. In light of the growing recognition of the interplay between chronotype, dietary behaviors, and cardiometabolic risk, it is scientifically reasonable to propose an integration of the MD framework, such as the pyramid recently proposed by SINU [51], by incorporating circadian and chronotype-specific considerations.

On a speculative level, a chronotype-oriented MD pyramid would not only stratify food groups according to frequency of consumption but also integrate recommendations regarding the temporal distribution of energy and nutrients. In this context, the principles of chrononutrition are central: the timing of meals regulates the synchronization of metabolic and hormonal rhythms (insulin, glucagon, cortisol, leptin among others), with implications for glucose homeostasis and metabolic homeostasis [191]. Evidence from TRE trials, where energy intake is confined to a 6–10-hour daytime window, demonstrates benefits on body weight, blood pressure, and glycemic control [192], supporting the incorporation of temporal guidance into the MD framework.

Similarly, chronotype emerges as a determinant of adherence to the MD [35, 47, 48]. Morning chronotypes show significantly higher adherence to the MD compared to evening chronotypes, whereas evening chronotypes more frequently delay meals, skip breakfast, and prefer energy-dense foods later in the day [35, 47, 48].

From a conceptual standpoint, morning chronotypes, characterized by earlier peaks in insulin sensitivity and energy expenditure [193], could benefit from a dietary pattern that concentrates caloric intake in the earlier part of the day. In this context, the MD may be structured to emphasize a nutrient-dense breakfast and a substantial midday meal, while recommending a light evening meal with a predominance of vegetables, legumes, and lean proteins. Such a distribution would maximize metabolic efficiency.

In contrast, evening chronotypes tend to exhibit delayed meal timing, reduced morning appetite, and a greater propensity toward late-night energy intake, which have been associated with impaired glucose metabolism and higher cardiometabolic risk [193–196]. For these individuals, a Mediterranean-oriented plan should prioritize gradual realignment of eating patterns toward earlier hours, while maintaining the qualitative aspects of the MD. Strategies might include reinforcing lunch as the principal meal of the day, moderating evening caloric load with easily digestible foods, and reducing nocturnal snacking by encouraging the intake of non-caloric beverages or low-energy Mediterranean staples such as herbal infusions or fruit. Additionally, evening meals could emphasize foods that support sleep quality, such as those rich in tryptophan (e.g., dairy products, nuts, seeds), melatonin-containing foods (e.g., cherries, grapes), and complex carbohydrates that may enhance serotonin synthesis and facilitate sleep onset, aligning both metabolic and circadian benefits [197]. For intermediate chronotypes, whose circadian profiles are less polarized, a balanced distribution of energy across the three main meals may be appropriate, while gradually favoring patterns observed in morning chronotypes.

Sleep quality and duration must also be considered as essential determinants within this model. Adequate sleep, defined not only by duration but also by continuity and architecture, exerts profound effects on appetite-regulating hormones (e.g., leptin, ghrelin), glucose metabolism, and cardiovascular homeostasis [198]. Short or fragmented sleep has been linked to increased appetite for calorie-dense foods, reduced insulin sensitivity, and greater cardiometabolic risk [47, 199]. Conversely, maintaining sufficient and regular sleep patterns enhances dietary self-regulation, improves adherence to healthy dietary patterns such as the MD, and protects against circadian misalignment. Sleep disturbances, including insomnia, social jetlag, and sleep-disordered breathing, represent critical barriers to effective implementation of the MD and should therefore be addressed as lifestyle priorities in parallel with dietary guidance [200].

**Fig. 2:**
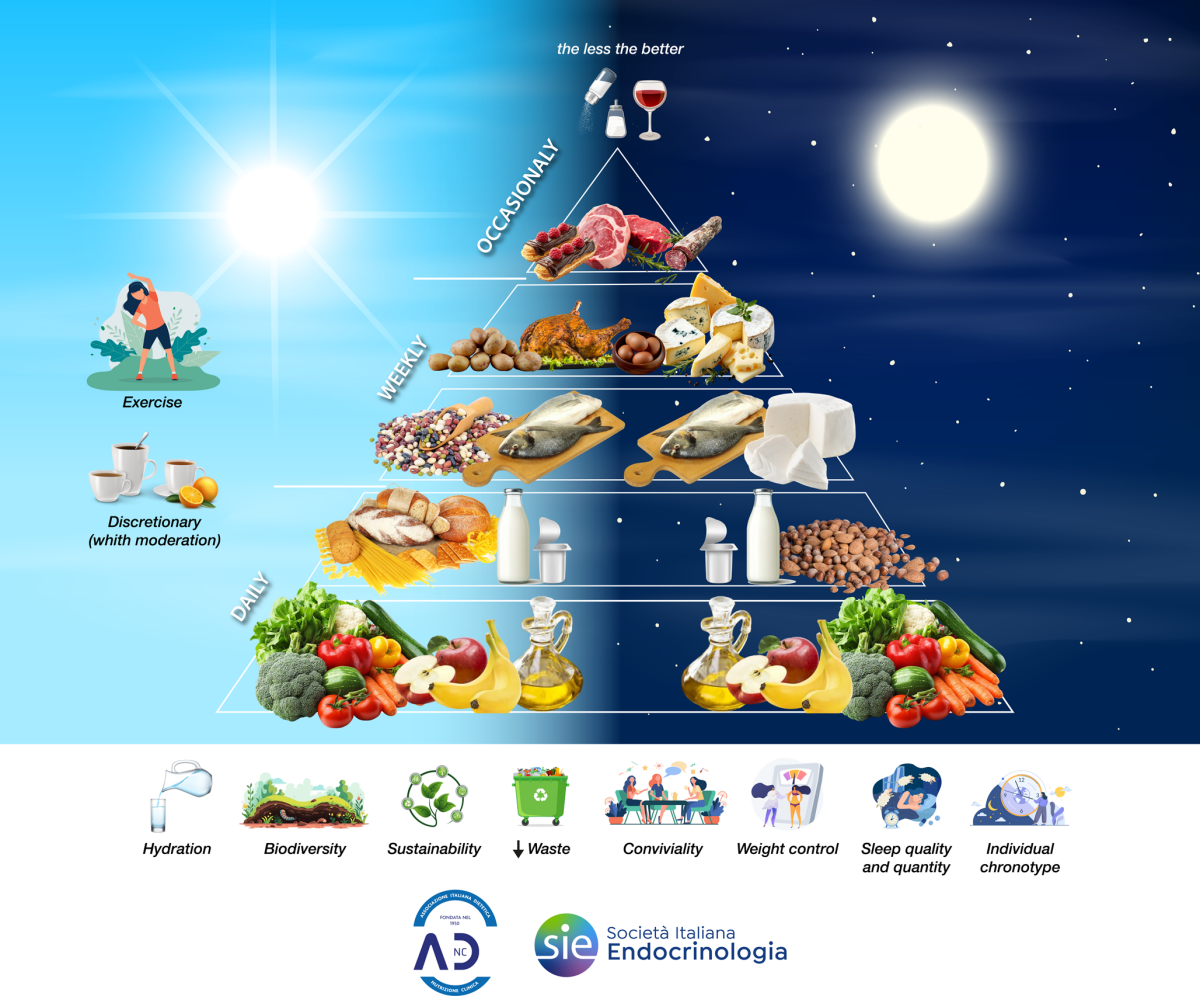
Chronotype-Based Mediterranean Diet Pyramid of the Italian Society of Endocrinology (SIE) and the Italian Society of Dietetics and Clinical Nutrition (ADI). Graphical representation of the Mediterranean Diet (MD) pyramid updated according to chrononutrition principles and the most recent LARN 2024 and SINU recommendations. The model preserves the traditional hierarchical structure of foods while incorporating circadian timing cues, depicted by sun (☀) and moon () symbols, to indicate the optimal timing of food consumption across the day

Taken together, these findings support the development of a novel MD pyramid that integrates chronobiological principles (Fig. 2). Beyond food frequency and quantity, the circadian-oriented MD pyramid should also be interpreted through the lens of nutrient timing, aligning intake with chronotype-specific and circadian considerations, in line with the most recent LARN 2024 and SINU recommendations (Table 2).

**Table 2:**
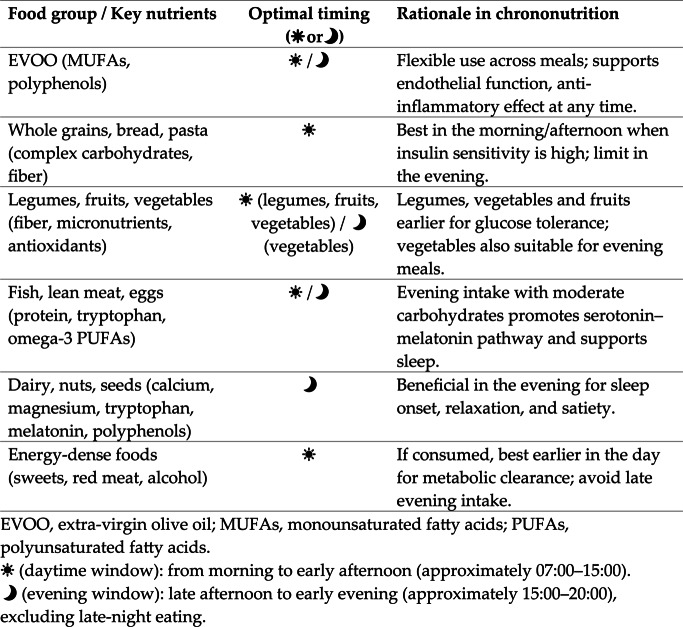
Nutrient timing within the circadian-oriented Mediterranean Diet pyramid (adapted from LARN 2024 and SINU guidelines)

The updated model retains the traditional hierarchical representation of foods but incorporates circadian cues, represented by “sun” (☀) and “moon” symbols, to indicate optimal timing of consumption.

- EVOO: as the primary source of MUFAs and bioactive polyphenols, EVOO is recommended for daily consumption. Given its metabolic neutrality and benefits on endothelial function and inflammation, EVOO can be incorporated flexibly across the day (☀/), supporting both morning and evening meals.
- Whole grains, bread, and pasta: as carbohydrate-rich foods, their timing is critical. In line with circadian glucose tolerance, these foods are best consumed earlier in the day (☀), where insulin sensitivity is greatest, while evening intake should be moderated to avoid late glycemic excursions.
- Legumes, fruits, and vegetables: rich in fiber, complex carbohydrates, antioxidants, and micronutrients, these foods may be distributed across the day, with carbohydrate-rich legumes and fruits preferentially consumed at breakfast and lunch (☀). Vegetables, particularly those rich in magnesium, potassium, and bioactive compounds, can also be emphasized in evening meals  for their favorable impact on digestion, satiety, and cardiometabolic health.
- Fish, lean meats, and eggs: high-quality protein sources may be included at both midday and evening meals. Evening intake can be particularly beneficial when combined with moderate amounts of carbohydrates, as protein-derived tryptophan requires an insulin-mediated context to cross the blood-brain barrier, thereby promoting serotonin and melatonin synthesis and supporting sleep quality.
- Dairy products, nuts, and seeds: as sources of calcium, magnesium, tryptophan, and bioactive peptides, these foods are particularly relevant in the evening , where they can enhance sleep onset and continuity. Nuts and seeds also provide melatonin and polyphenols, aligning circadian and metabolic benefits.
- Energy-dense foods (sweets, red meat, alcohol): consistent with the MD principles and LARN 2024 recommendations, these foods should be limited. If consumed, they are best confined to earlier hours of the day (☀), when metabolic clearance is more efficient, reducing the risk of nocturnal glycemic and lipid disturbances.

Finally, at the base of the pyramid, lifestyle determinants such as regular physical activity, adequate and high-quality sleep, and the alignment of meal timing with individual chronotype are emphasized as non-nutritional but essential foundations of the Mediterranean lifestyle.

## Conclusions

The MD remains one of the most evidence-based nutritional models for the prevention of non-communicable diseases, but its implementation has traditionally overlooked the role of circadian biology and chronotype. Integrating principles of chrononutrition into the MD framework highlights the importance of not only what is eaten but also when foods are consumed. By tailoring food timing, meal frequency, and nutrient distribution to individual chronotypes, the proposed circadian-oriented MD pyramid provides a more personalized and biologically consistent approach (Box 2).

**Box 2** Chronotype-adapted Mediterranean Diet: practical recommendations for clinicians

**Table:** 

• Assess chronotype using brief validated tools (e.g., MEQ, rMEQ, CSM, MCTQ) to determine morning, intermediate, or evening preference. • Prioritize early energy intake for all chronotypes, especially evening types: encourage a nutrient-dense breakfast and a substantial midday meal. • Time carbohydrates earlier in the day when insulin sensitivity is highest; limit refined and starchy foods in the evening. • Favor vegetables and lean proteins at dinner to support satiety, digestion, and sleep quality. • Support sleep-promoting foods in the evening such as dairy, nuts, seeds, and tryptophan-rich protein sources. • Progressively shift late eaters toward earlier meal timing using small, feasible adjustments (e.g., advancing dinner by 15–20 min weekly). • Anchor eating windows by setting consistent mealtimes aligned with the individual’s circadian profile; avoid nocturnal snacking • Address social jetlag by stabilizing sleep–wake rhythms, reducing evening screen use, and reinforcing morning light exposure. • Integrate physical activity preferably in daylight hours to strengthen circadian alignment and improve metabolic flexibility. • Reinforce core MD principles (high plant-food intake, EVOO as main fat, limited red/processed meat) while adapting nutrient timing rather than changing food quantity.	

This model emphasizes early intake of carbohydrate-rich foods, evening preference for vegetables and proteins, and the inclusion of sleep-promoting nutrients, alongside lifestyle determinants such as adequate sleep and physical activity. Beyond nutritional guidance, this approach redefines the MD as a holistic lifestyle paradigm that harmonizes diet, circadian rhythm, and sleep hygiene. Future research and clinical applications should evaluate its feasibility, long-term adherence, and impact on metabolic and cognitive health, thereby consolidating the role of the MD as both a cultural heritage and a modern, chronobiology-informed public health strategy. 

## Key References

- Sofi F, Martini D, Angelino D, Cairella G, Campanozzi A, Danesi F, Dinu M, Erba D, Iacoviello L, Pellegrini N, Rossi L, Vaccaro S, Tagliabue A, Strazzullo P. Mediterranean diet: why a new pyramid? An updated representation of the traditional Mediterranean diet by the Italian Society of Human Nutrition (SINU). Nutr Metab Cardiovasc Dis. 2025;35:103919.
  - ○ This updated representation of the Mediterranean Diet pyramid by SINU redefines its components for contemporary lifestyles, forming the scientific basis for adaptations such as the chronotype-tailored model proposed by SIE and ADI.
- Hu FB, Drescher G, Trichopoulou A, Willett WC, Martínez-González MA. Three decades of the Mediterranean diet pyramid: a narrative review of its history, evolution, and advances. Am J Clin Nutr. 2025;122:17–28.
  - ○ This narrative review traces the evolution of the Mediterranean Diet pyramid, emphasizing its ongoing adaptation toward sustainability, personalization, and health optimization—core principles of the new chronotype-based model.
- Zinna L, Verde L, Tolla MF, Barrea L, Parascandolo A, D’Alterio F, Colao A, Formisano P, D’Esposito V, Muscogiuri G. Chronodisruption enhances inflammatory cytokine release from visceral adipose tissue in obesity. J Transl Med. 2025;23:231.
  - ○ The study links circadian misalignment to increased inflammatory signaling in adipose tissue, reinforcing the importance of circadian-friendly dietary patterns like the Mediterranean Diet for metabolic health.
- Van der Merwe C, Münch M, Kruger R. Chronotype differences in body composition, dietary intake and eating behavior outcomes: a scoping systematic review. Adv Nutr. 2022;13:2357–405.
  - ○ This systematic review provides compelling evidence of how chronotype influences dietary behavior, energy intake, and body composition, underscoring the rationale for aligning Mediterranean Diet recommendations with individual circadian profiles.

## Data Availability

No datasets were generated or analysed during the current study.
